# Post-traumatic stress disorder and associated factors among high school students who experienced war in Woldia town

**DOI:** 10.3389/fpsyt.2024.1359370

**Published:** 2024-07-16

**Authors:** Mulat Awoke Kassa, Sefineh Fenta, Tamrat Anbesaw, Natnael Amare Tesfa, Alemu Birara Zemariam, Genanew Mulugeta Kassaw, Biruk Beletew Abate, Elsabet Gezmu Semagn

**Affiliations:** ^1^ Department of Nursing, College of Health Sciences, Woldia University, Woldia, Ethiopia; ^2^ Department of Public Health, College of Health Sciences, Woldia University, Woldia, Ethiopia; ^3^ Department of Psychiatry, College of Medicine and Health Sciences, Wollo University, Dessie, Ethiopia; ^4^ School of Medicine, College of Health Sciences, Woldia University, Woldia, Ethiopia; ^5^ Department of Pediatrics and Child Health Nursing, School of Nursing, College of Medicine and Health Science, Woldia University, Woldia, Ethiopia; ^6^ Department of Veterinary Parasitology, School of Veterinary Medicine, Wollo University, Dessie, Ethiopia

**Keywords:** post-traumatic stress disorder, high school students, adolescents, war, conflict, Ethiopia

## Abstract

**Background:**

The experience of war in recent time is very common around the world, and the impact is profound on the mental health of the victims, especially among the young population. The most implicated mental health problem is post-traumatic stress disorder, which comes after an exposure to trauma as a severe and long-term result of the traumatic event. Studies in developed countries revealed this finding, but there is insufficient information in developing countries, where much of war and conflict exist and young population live including Ethiopia. Therefore, this study aims to assess the prevalence and associated factors of post-traumatic stress disorder among high school students who experienced war.

**Objective:**

We assessed the prevalence and factors associated with post-traumatic stress disorder among high school students who experienced war.

**Methods:**

A multi-centered school base cross-sectional study was conducted from May 23 to June 08, 2022. Data were collected from high school students in Woldia town. Bivariable and multivariable logistic regression was used to identify the independent factors associated with post-traumatic stress disorder.

**Results:**

A total of 338 of the 410 students participated in this study (94.5% response rate). The prevalence of post-traumatic stress disorder was 39.2%. In the multivariable analysis, poor social support (AOR = 3.40, 95% CI: 1.45, 7.95), depression (AOR = 3.24, 95% CI: 1.69,6.21), high level of perceived stress (AOR = 2.98, 95% CI: 1.61, 5.50), being in war fighting situation (AOR = 2.85, 95% CI: 1.40, 5.78), and witnessing the murder of family members or friends (AOR = 3.05, 95% CI: 1.47, 6.32) were factors significantly associated with post-traumatic stress disorder at a *p*-value <0.05.

**Conclusions and recommendations:**

In this study, around two in five of high school students had post-traumatic stress disorder. Independent factors of PTSD were depression, high stress levels, poor social support, witnessing the murder of family members/friends, and being in war fighting situation. We recommend that the Ministry of Education and the Ministry of Health collaborate to integrate mental health services into schools. This focuses on the early detection of students at risk of PTSD, such as those with depression, high perceived stress levels, and exposure to murder or war, and provides necessary social support to prevent PTSD.

## Introduction

Adolescence is a time period between childhood and adult. Any person between the ages of 10 and 19 is considered as an adolescent by the World Health Organization (WHO) (1). Because adolescence constitutes a unique stage of development, this population group is particularly susceptible to certain threats and risks that create specific needs and greater vulnerability (2). The WHO states that half of all mental health disorders that are fully developed during adulthood begin before the age of 14 (3).

Post-traumatic stress disorder (PTSD) is one of the trauma and stress-related disorders that develop after experiencing or witnessing a stressful, frightening, or life-threatening event like combats, disasters, or sexual assaults (4). Individuals with PTSD suffer from loneliness, irritability, guilt, difficulty concentrating, difficulty sleeping, and dreams or flashbacks that bring back the horrific incident. They also attempt to avoid circumstances, people, and places that remind them of the traumatic event (5). In general, the symptoms of PTSD in adolescents are similar to the symptoms of PTSD in adults (6). Adolescents may show specificities with extreme reactions than in adults such as becoming more aggressive, difficulty of controlling their impulses, and substances misuse (7). In addition, adolescents frequently experience nightmares, emotional insensitivity, conscious avoidance of anything that could trigger memories of the traumatic event, as well as frequent depression, antisocial behavior, physical complaints, decreased academic performance, sleeping problems, and suicidal thoughts (8).

Armed conflict, war, or insurgency that affects civilian populations increased toward the end of the 20th century. At this time, violence and attacks against civilian targets are common in wars around the world, which lead to population danger, insecurity, and anxiety. More than a billion youth worldwide live in countries where an armed conflict, war, or terrorism is common (9). In Ethiopia, Tigray People Liberation Front (TPLF) forces have been fighting an open assault against the Federal Republic Government of Ethiopia at Amhara and Afar regions since June 2021, leaving the population there in terrible economic and social circumstances. The invading force targeted a number of rural farmers, city people, teachers, and medical professionals who were not politically engaged (10). Children and adolescents are the most at risk of suffering the effects of these hostilities because they must negotiate the tasks of normal developmental growth against a backdrop of insecurity and violence, which increases their vulnerability and susceptibility (11).

After trauma, up to 36% of adolescents exposed to trauma have a risk of developing PTSD (11). A meta-analysis study from North America, Europe, Australia, and Asia revealed that approximately 16% of trauma-exposed adolescents had PTSD (12). A recent systematic review and meta-analysis study among adolescents in low- and middle-income countries (LMICs) reported that the prevalence of PTSD range from 0.2% to 87% (13). According to a meta-analysis of studies published from 1998 to 2011, the prevalence of PTSD among adolescents exposed to a traumatic event was 15.9% (12). Another meta-analysis study done among adolescents showed that the prevalence of PTSD was 19.9% after road traffic accidents (14). In addition, studies done among adolescents reported that the prevalence of PTSD after an earthquake was 58.3% in Indonesia, 66.7% in Iran, and 43.3% in Nepal (15–17). Furthermore, other studies done among school students showed that the prevalence of PTSD was 26.8% in Kenya (18), 16.4% in Palestine (19), 31% in Jordan of Syrian refuge (20), 53% in Syrian (21), 19.3%, 63.9%, and 70.4% in Morocco studies (22–24), 46.3% in China (25), 60% in Uganda (26), and 61% in Iraq (27). Some studies from Ethiopia showed that the prevalence of PTSD among individuals in war-affected areas was 59.8% in Northwest Ethiopia (28), 58.4% in South Ethiopia (29), 17.1% in West Ethiopia (30), 37.3% in Addis Ababa (31), 19.4% in Dessie town (32), and 56.28% in Woldia town (33).

Different variables can affect the prevalence of PTSD among adolescents as studies have reported. These variables—which are categorized as variables that occurred prior to the traumatic event, such as female sex (29, 34, 35), younger age, poor social support, chronic medical illness, drinking alcohol (31, 36–44), and a previous history of mental illness like depression symptoms (28, 29, 43, 45); factors which occurred during traumatic events, like witnessing the death of a family member or friends (29, 36, 46–49), type of trauma, and serious physical injury (50–52); and variables which occurred following traumatic events, for example, having a high level of perceived life stress and destruction of property—were the predictors of PTSD (28, 31, 53–55). 

The effect of PTSD on school-age adolescents is profound as it will lead to low self-esteem, alcoholism, and other problems such as substance abuse, poor academic performance, poor relationship with family members, and even social isolation and self-harming behaviors (52, 56, 57). Therefore, in order to prevent more suffering, post-traumatic stress disorder symptoms need to be handled as soon as they arise (15). Even if Ethiopia is experiencing different conflicts recently, nearly around all regions, but limited studies were done on the impacts of these conflicts on the development of post-traumatic stress disorder, especially on the mental health of adolescents. Therefore, the aim of this study was to assess the level of post-traumatic stress disorder and its predictors among high school students. The research questions are as follows: 1. How much is the magnitude of PTSD among high school adolescents who experienced war? 2. Which factors are affecting the magnitude of PTSD among high school adolescents who experienced war?

## Materials and methods

### Study area and populations

In May 2022, a multi-centered, school-based, cross-sectional study was carried out at four high schools in Woldia town, Northeastern Ethiopia. The schools are Mesenado Secondary and Preparatory School, Millennium Secondary and Preparatory School, Genetie Secondary and Preparatory School, and Woldia Secondary and Preparatory School (58). The study area is located 521 kilometers from Addis Ababa, the capital city of Ethiopia, in the North Wollo Zone of the Amhara National Regional State. In the study area, there were 46,139 residents overall, including 23,000 men and 23,139 women. Moreover, 80.49% of the total residents are Ethiopian Orthodox Christians religion followers, while 18.46% are Muslims and the rest are Protestants. This study included all secondary and preparatory school students who had attended their classes during the period of data collection and had lived in the study area at least for the last preceding year of the war. Students who were unable to communicate, those who were acutely sick during the time of data collection, and students who lived in the study area for less than a year during the conflict were excluded from the study (58).

### Sample size determination

The sample size was determined by taking a single population proportion formula assumption. We calculated the sample size using the proportion from a previous study with proportion (p) of 58.4% (29), with 95% confidence interval (CI) and margin of error 5%, and 10% non-response rate. Then, the final sample size was 410.

### Student recruitment procedures

Before the actual data collection time, the students were first stratified by their grade level as grade 9, grade 10, grade 11, and grade 12, considering each grade level as strata. The data we get on education of the study region showed that there were a total of 5,100 high school students (58). Among these, grade 9 accounts for 1,606, grade 10 accounts for 1,230, grade 11 accounts for 1,179, and grade 12 accounts for 1,085 of the students from the total number of high school students. Then, we made a proportional allocation for each stratum (grade levels), and as a result, 129 students from grade 9, 99 students from grade 10, 95 students from grade 11, and 87 students from grade 12 were drawn. Finally, a computer-generated lottery method using the students’ identification number was applied to select the study participants from each stratum. In the end, the selected students in each stratum were taken to one hall, which has a separate entry and exit for the study participants, and each study participant was entered and filled the questionnaire and went out through the exit of the hall without contacting another study participant to ensure no information sharing and secure their anonymity. Then, the questionnaires were administered after orientation through direct contact with the study participants but keeping their anonymity, and all the filled questionnaires were collected by the data collectors.

### Measurement and data collection

PTSD was measured by using a 20-item Post-Traumatic Checklist (PCL-5) with scores ranging from 0 to 80 with a five-point Likert scale (0 = not at all, 1 = a little bit, 2 = moderately, 3 = quite a bit, 4 = extremely). The participants were asked based on their experiences over the last month. Then, summing up all points, those participants scoring ≥33 were considered as having PTSD. PCL-5’s validity and reliability have been examined and demonstrated and were used to assess PTSD. PCL-5 was used and validated in Rwanda students to have internal consistency (Cronbach alpha) of 0.934, sensitivity of 88.7%, and specificity of 88.9% (59). The internal consistency (Cronbach alpha) of PCL-5 in this study was 0.89. In the present study, the nine-item Patient Health Questionnaire (PHQ-9), with scores ranging from 0 to 27 with four-point Likert scale (0 = not at all, 1 = several days, 2 = more than half the days, 3 = nearly every day), was used to assess depression, with a score of 10 or higher indicating depression. The participants were asked based on their experiences over the past 2 weeks. PHQ-9 was validated in Nigerian students, and the internal consistency (Cronbach alpha) was 0.85, the sensitivity was 87.9%, and the specificity was 98.9%, respectively (60). The internal consistency (Cronbach alpha) of (PHQ-9) in this study was 0.84.

Perceived life stress was assessed using Perceive Stress Scale (PSS) scale, which is widely used as a psychological instrument to measure the perception of stress. PSS has a five-point Likert scale (0 = never, 1 = almost never, 2 = sometimes, 3 = fairly often, 4 = very often) with 0–40 range. The participants were asked based on their experiences over the last month. Respondents with scores between 0 and 13 were considered to have a low level of perceived stress, those between 14 and 26 were considered to have a moderate level of perceived stress, and participants who scored between 27 and 40 were considered to have a high level of perceived stress. A study done in Bhutan showed the internal consistency of this PSS as 0.90 (61). The internal consistency (Cronbach alpha) of Perceived Stress Scale (PSS) in this study was 0.82.

The three-item Oslo (Oslo-3) tool was used to assess the level of social support of the participants with a score range of 3–14. Participants with a score of 3–8 were categorized as having a poor social support level, those with a score of 9–11 were categorized as having a moderate social support level, and participants with a score between 12 and 14 were categorized as having a strong social support level (62). A study done in Nigeria showed that the internal consistency (Cronbach alpha) of Oslo-3 social support was 0.5 (63), and the internal consistency (Cronbach alpha) of Oslo-3 social support in this study was 0.79.

Substance use history of the participants including Khat, tobacco, and alcohol was assessed using yes/no (yes = 1, no = 0) questionnaires adapted from the ASSIST (Alcohol, Smoking, and Substance Involvement Screening Test) (64). The questions include about ever substance use history as well as current substance use history. Ever substance use history asks about any history of substance use in the individual’s life, and current substance use history asks about history of substance use in the past 3 months. Anxiety was assessed using GAD-7, which is a self-administered questionnaire designed for screening and measuring the severity of generalized anxiety disorder. GAD-7 consists of seven items that respondents respond to base on their experiences over the past 2 weeks. GAD-7 had a four-point Likert scale, with each item scored from 0 (not at all) to 3 (nearly every day) and with the score ranging from 0 to 21; individuals who scored 10 and above were considered as having anxiety (65). GAD-7 has a sensitivity of 89% and a specificity of 82% (66). The internal consistency of GAD-7 in this study was 0.90. Socio-demographic characteristics such as age, sex, and grade level were collected by using structured socio-demographic questionnaires (sex of participants: male = 1, female = 0, urban residence = 1, rural residence = 0). Factors like family history of mental illness, trauma-related factors, and history of chronic medical illness were assessed using a structured yes/no (yes = 1, no = 0) questionnaire.

The collected data were coded, edited, entered, and checked into the computer using EPI data version 4.6.02 and imported to STATA version 14.0 to generate descriptive statistics like means, standard deviation, frequency, and percentages. To determine an association between dependent and independent variables, adjusted odds ratios were used using logistic regression, and the significance level was determined using a confidence interval of 95%. Bivariable and multivariable logistic regression, respectively, was used to identify the independent predictors of PTSD. Each independent variable was separately entered in the bivariable analysis. Then, variables with a *p*-value <0.2 on bivariable analysis were entered into multivariate analysis. Then, variables that showed a statistically significant association with a *p*-value <0.05 on multi-variable analysis were considered to be the predictors of PTSD.

### Statistical analysis

#### Parametric properties of data collection tools

As checked by Shapiro–Wilk test for PCL-5 (*p* = 0.39), PHQ-9 (*p* = 0.25), PSS (*p* = 0.48), Oslo-3 (*p* = 0.65), and GAD-7 (*p* = 0.92), the revealed *p*-values were insignificant for each tool; this indicated a normal distribution. Because the data were normally distributed, we calculated the mean for each independent variable: PCL-5 (mean = 29.6, standard deviation = 2.35), PHQ-9 (mean = 11.5, standard deviation = 1.99), PSS (mean =25.4, standard deviation = 2.21), Oslo-3 (mean = 9.405, standard deviation = 2.54), and GAD-7 (mean = 10.3, standard deviation = 2.35). The skewness test of these tools indicated the following: PCL-5 = 0.0730, PHQ-9 = 0.078, PSS = 0.060, Oslo-3 = 0.62, and GAD-7 = 0.082. Multi-collinearity was assessed for the independent variables, and VIF was less than 10.

#### Data quality control

To control the quality of data, the questionnaire was translated appropriately into the local Amharic language with a local language expert. The training was given to data collectors and supervisors, and each completed questionnaire was checked and the necessary feedback was also offered to data collectors each following morning. The questionnaire was pretested 1 week before the actual data collection time on 5% (*n* = 21) of the study participants who were not included in the main study.

## Results

### Socio-demographic characteristics of participants

Data were obtained from 388 high school students, with a response rate of 94.5%. The mean age of the participants was 17.82 ± 1.66, ranging from 14 to 25 years old, and the majority of the ages of 48.2% of the participants was ranging between 14 and 18 years old. More than half of the number (55.67%) of the participants were male, and 69.59% of them were from urban areas, while the rest were from rural areas as shown in **Table 1**.

**Table 1:** Socio-demographic characteristics of the study participants.

Variables	Categories	Frequency	Percent
**Age**	<18	187	48.2
	=18	63	16.2
	≥18	138	35.6
**Sex**	Male	216	55.7
	Female	172	44.3
**Residency**	Rural	118	30.4
	Urban	270	69.6
**Grade level**	9	160	41.2
	10	109	28.1
	11	62	16.0
	12	57	14.7
**Semester average score in percentage**	<70%	226	58.3
	70%–84.5%	138	35.6
	≥85%	24	6.1

### Clinical characteristics of the respondents

**Figure 1:**
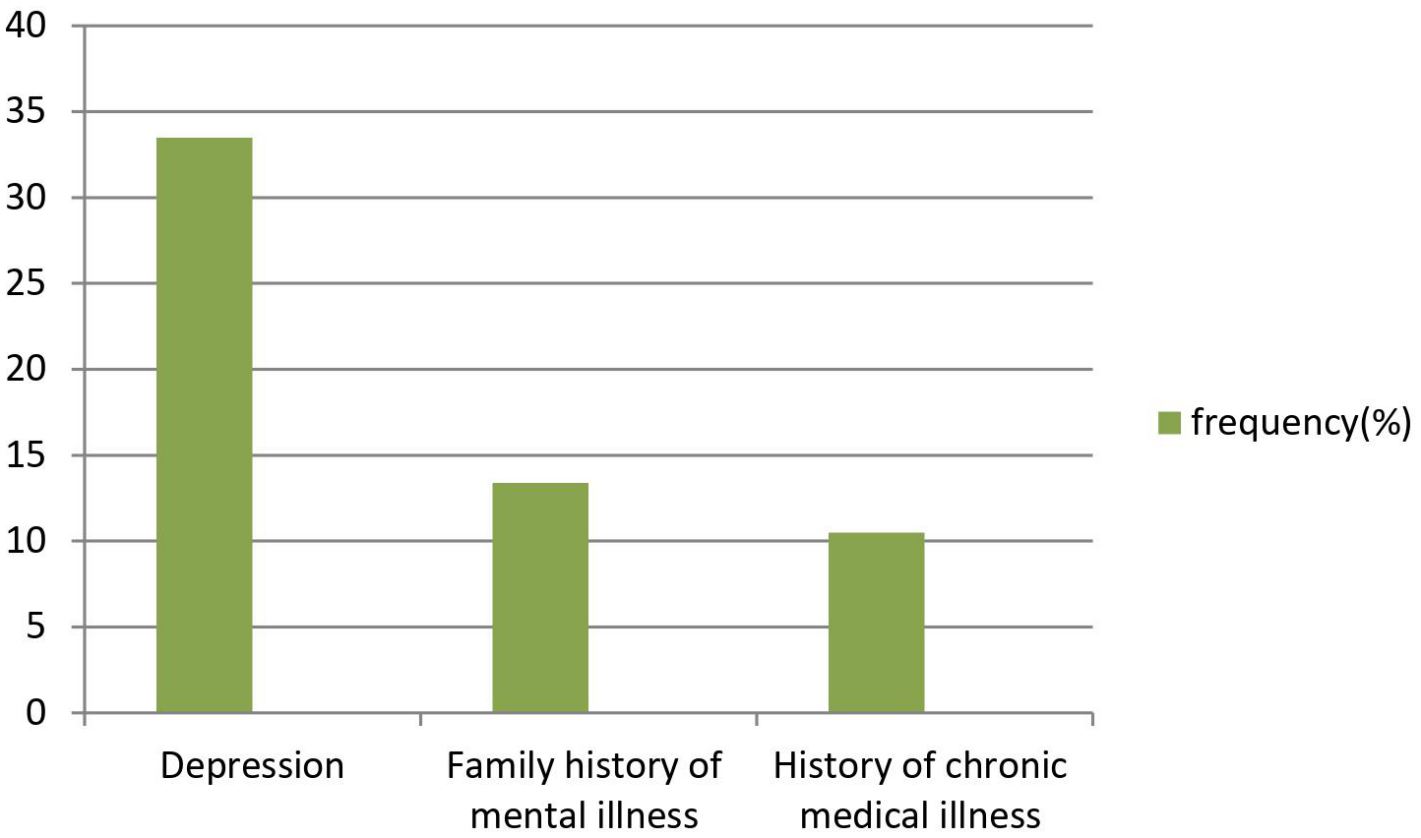
Shows clinical characteristics of study participants.

Out of the total participants, 10.57% of students had a history of chronic medical illness, 13.40% of students had a family history of mental illness, and 33.51% had depression as seen in **Figure 1**.

### Substance-related characteristics of the participants

**Figure 2:**
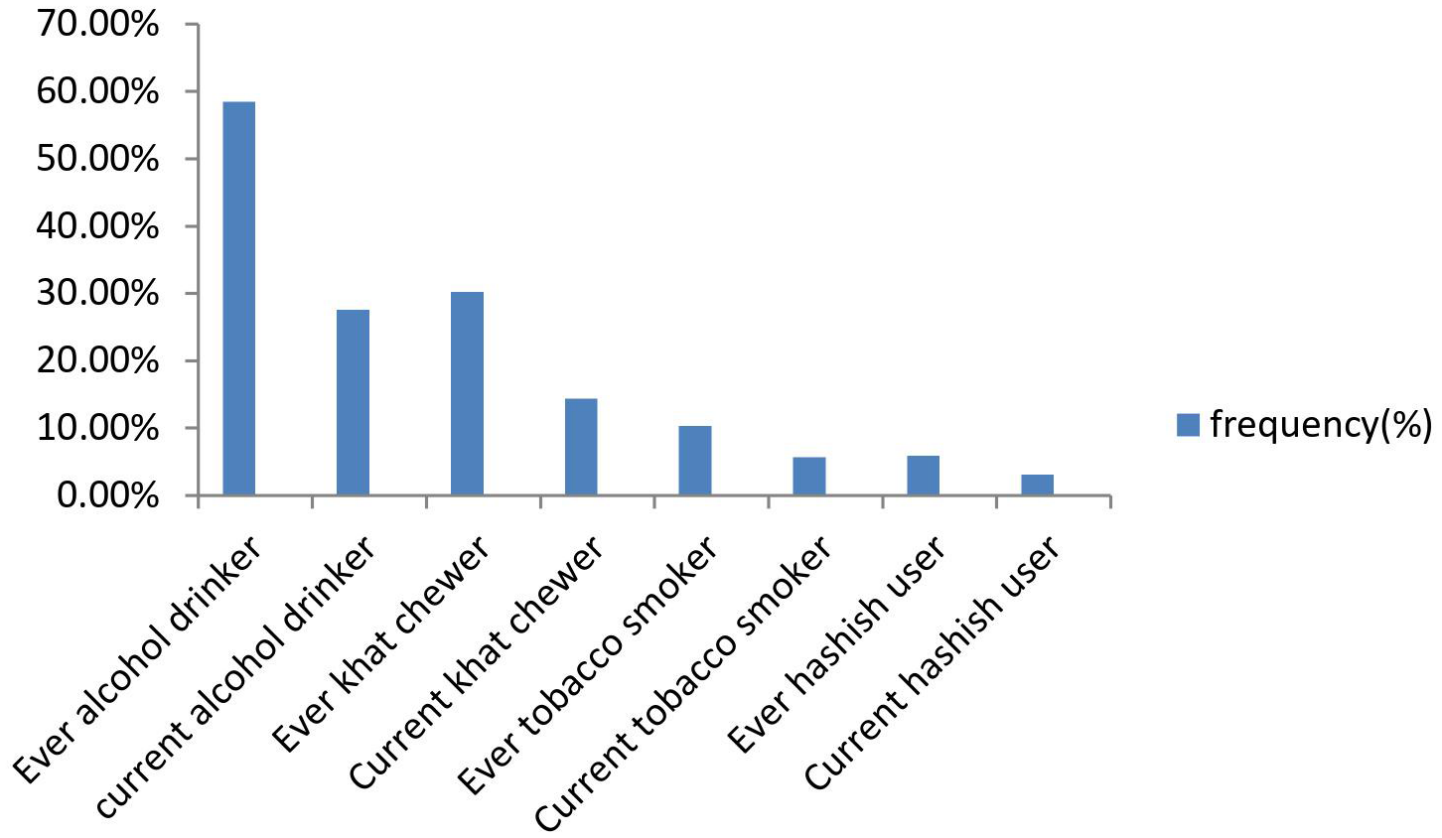
Substance-related characteristics of the study participants.

Regarding substance use, out of the total participated students, 58.5% were ever alcohol drinkers, whereas Khat and cigarette ever users were 30.2% and 10.3%, respectively, and 27.6% of them were current alcohol drinkers as shown in **Figure 2**.

### Individual trauma type and psychosocial-related characteristics of participants

Among the study participants, 52.8% were in a war fighting situation, 16.5% had a destruction of property, 19.1% witnessed the murder of family or friends, 16% had ill health without medical care, 13.1% had a strong social support, 28.6% had a moderate social support, and 58.3% had a poor social support, and 37.1%, 20.9%, and 42.0%, of the respondents had a high, moderate, and low level of perceived stress, respectively.

### Prevalence and associated factors of post-traumatic stress disorder

In this study, the overall prevalence of post-traumatic stress disorder among high school students was 39.2% (95% CI: 34.4, 44.1). Having depression, high and moderate levels of perceived stress level, witnessing the murder of family or friends, having ill health without medical care, being tortured or beaten, witnessing the murder of a stranger, imprisonment against will, forced separation from the family, having moderate and poor social support levels, and being in a war fighting situation were factors associated with post-traumatic stress disorder at *p* < 0.2 in binary logistic regression.

Finally, in the analysis of multivariable logistic regression model, this study showed that having depression, high and moderate levels of perceived stress level, witnessing the murder of family members or friends, being in a war fighting situation, and having poor social support were found to be significantly associated with post-traumatic stress disorder, with 95% of CI and at *p*-value <0.05 as shown in **Table 2**.

**Table 2:** Bivariable and multivariable analysis of factors associated with post-traumatic disorder among high school students in Woldia town, 2022 (*n* = 388).

	Category	Post-traumatic stress disorder		COR (and 95% CI)	AOR (and 95% CI)
**Variables**		Yes	No		
**Depression**	Yes No	92 60	38 198	7.98 (4.96–12.85)	3.24 (1.69– 6.21)***
**Perceived stress level**	High Moderate	70 32	74 49	2.13 (1.34–3.40) 1.47 (0.84–2.57)	2.98 (1.61–5.50)*** 2.45 (1.18–5.06)*
**Witnessing murder** **of family/friends**	Yes No	53 99	21 215	5.48 (3.13–9.58)	3.05 (1.47–6.32)**
**Ill health without** **medical care**	Yes No	31 121	31 205	1.69 (0.98–2.92)	0.75 (0.36–1.57)
**Social support level**	Poor Moderate	124 16	102 95	3.95 (1.96–7.94) 0.54 (0.23–1.26)	3.40 (1.45–7.95)** 0.60 (0.22–1.62)
**Being tortured/beaten**	Yes No	31 121	35 201	1.470 (.86–2.50)	0.68 (0.33–1.41)
**Having witnessing** **murder of stranger**	Yes No	66 86	41 195	3.65 (2.29–5.81)	1.53 (0.78–2.99)
**Imprisonment against one’s will**	Yes No	35 117	38 198	1.55 (0.93–2.60)	0.72 (0.36–1.44)
**Forced separation** **from the family**	Yes No	52 100	24 212	4.59 (2.67–7.87)	1.03 (0.49–2.16)
**Being in war fighting situation**	Yes No	119 33	86 150	6.28 (3.93–10.04)	2.85 (1.40–5.78)**

*p-value < 0.05; **p-value < 0.01; ***p-value < 0.001.

Students who had poor social support were 3.4 times more likely to have post-traumatic stress disorder compared to students who had a strong social support (AOR = 3.40, 95% CI: 1.45, 7.95). Students who witnessed the murder of family members or friends were about 3.1 times more likely to have post-traumatic stress disorder than students who did not witness the murder of family members/friends (AOR = 3.05, 95% CI: 1.47, 6.32).

Students who were in a war fighting situation had 2.9 times higher post-traumatic stress disorder than students who were not in a war fighting situation (AOR = 2.85, 95% CI: 1.40, 5.78). Students who had depression reported 3.2 times higher post-traumatic stress disorder than students who had not depression (AOR = 3.24, 95% CI: 1.69, 6.21). Students with a high level of perceived stress level had three times higher post-traumatic stress disorder than students who have a low level of perceived stress (AOR = 2.98, 95% CI: 1.61, 5.50).

## Discussion

The findings of the current study showed that the prevalence of post-traumatic stress disorder among high school students in Woldia town was 39.2% (95% CI: 34.4, 44.1), which is similar with studies done in Palestine, Turkey, Nepal, and Ethiopia with a reported prevalence of 36%, 40.6%, 43.3%, and 36.5%, respectively (67–69).

However, the prevalence of post-traumatic stress disorder in this study was higher than studies done in Ukraine 28% (48), Jordan 31% (20), Kenya 26.8% (18), Saudi Arabia 24.8% (70), and Morocco 19.3% (22). The possible reason for this difference may be due to the difference in the instruments that they used—for example, a study done in Ukraine was conducted using the Harvard Trauma Questionnaire tool; in the Kenya study, UCLA PTSD-RI (UCLA PTSD Reaction Index) tool was used; and in the Jordan study, the Child Post-traumatic Stress Disorder Symptom Scale (CPSS) tool was used; whereas in this study, the PCL-5 with extended criteria A modified and having better internal consistency was used (71). Different studies evidenced that the difference in the instrument tool affects the prevalence of post-traumatic stress disorder (29, 31, 40). Another reason might be due to the difference in the timing of these studies when conducted after the study participants faced a traumatic event—for example, a study done in Ukraine was conducted 2 years after Russia invaded areas of Eastern Ukraine, a study done among Syrian refugees in Jordan was conducted after 3 years of conflict, but the current study was conducted 12 months after the participants experienced traumatic events. If studies are conducted long after the traumatic events, the participants will have a probability of decreased severity perception about the traumatic events due to recall bias, which may affect the participants’ report (72). Furthermore, the difference in the prevalence rate could be explained by the difference in trauma type and socio-cultural difference among study participants of these studies. On the other hand, the current prevalence was lower than the prevalence of studies done in China with 46.3% (25), Syria with 53% (21), and Uganda with 60% (26), respectively. The difference may be attributed to the varying proportions of study participants with a comorbid mental illness in these studies—for instance, in studies conducted in Uganda and Syria, 58% and 51.5% of the participants, respectively, had depression. In contrast, our study found that only 33.5% of the participants had comorbid depression. Various studies have shown that having comorbid depression can elevate the risk of developing post-traumatic stress disorder (28, 29, 73–75). Another reason for this discrepancy could be the differences in the assessment tools, study designs, and the type and degree of exposure experienced by the participants in these studies.

The odds of post-traumatic stress disorder were 3.3 times higher among high school students who had poor social support compared to those who had strong social support. This finding was supported by studies done in Ethiopia (31, 40), Nigeria (39),US (37), China (36), and UK (38). The reason for this finding may be due to the fact that individuals with poor social support may lack the emotional and practical assistance needed to cope with the aftermath of traumatic events. Without support from friends, family, or community, they are more likely to experience heightened levels of stress and anxiety, which can contribute to the development of PTSD. Another reason could be explained by the lack of social support which may be expressed through a perception of being unsupported, which can exacerbate feelings of isolation and helplessness. When individuals believe that they have no one to turn to, their ability to process and recover from traumatic experiences diminish, which increases the likelihood of PTSD (76, 77). The finding of this particular relationship between social support and the risk of PTSD underscoring the importance of fostering strong social support systems and supportive environment within schools and communities by promoting positive relationships among students, teachers, families, and community members is essential to mitigate the risk of PTSD. In addition, counseling services and mental health resources are offered to students, particularly those who may be at risk due to limited social support. This can include individual counseling, support groups, and access to mental health professionals.

The odds of post-traumatic stress disorder were three times higher among students who witnessed the murder of family members or friends compared to those who had not. Studies done in Ethiopia (29, 46), Canada (49), China (36), Ukraine (48), and Turkey (47) supported this finding. The reason for this finding may be due to the fact that witnessing the murder of a loved one is an extremely distressing and traumatic experience. The intensity and severity of this trauma can significantly impact the individual’s psychological well-being, increasing their vulnerability to PTSD. Another reason might be due to the fact that witnessing the murder of a family member or friend can shatter a person’s sense of safety and security in the world. This loss of trust in their environment and the people around them can contribute to the development of PTSD symptoms, as individuals may constantly feel on the edge or hyper-vigilant to potential threats. Moreover, this finding may be attributed to the intertwining of grief resulting from the loss of a loved one and trauma stemming from witnessing their murder. This combination creates a complex and lasting psychological burden, intensifying PTSD symptoms and complicating the healing and recovery process for individuals (78, 79). This implies offering immediate access to mental health professionals trained in trauma-focused therapy to provide support and intervention for students who have witnessed such traumatic events. Adopt trauma-informed approaches in schools, ensuring that all interactions and interventions are sensitive to the needs of students who have experienced trauma. This may involve modifying disciplinary practices, providing accommodations for academic performance, and creating a supportive school environment.

The odds of developing post-traumatic stress disorder (PTSD) were 3.5 times higher in students who had been in war fighting situations compared to those who had not. This finding was supported by studies done in Ethiopia (80), US (38), Iraq (81), and Turkey (82). The possible explanation for this finding could be that individuals in war zones are frequently exposed to highly traumatic events such as violence, death, and destruction. This exposure significantly increases their risk of developing PTSD (83). Another reason for this finding might be that persistent threat to life and the constant state of alertness required in war fighting situations can lead to chronic stress, which is a known contributor to PTSD (84). Additionally, the possible reason behind this finding might be that, during war situations, individuals often witness or directly experience severe violence, including injuries and deaths of fellow soldiers, civilians, and even family members. Such experiences can profoundly impact their psychological health (85). Furthermore, the chaotic nature of war can result in the loss of family members and friends, disrupting social support systems. The disintegration of these support networks leaves individuals more vulnerable to PTSD (86). Thus, it is essential to create a safe environment where affected students can share their experiences, feelings, and concerns with peers who have undergone similar trauma. Peer support can be invaluable in the healing process. In addition, it is imperative to teach students resilience-building skills and coping strategies to help them manage stress, regulate emotions, and navigate challenging situations. This may include mindfulness exercises, relaxation techniques, and problem-solving skills training.

Students with depression were 3.2 times more likely to develop post-traumatic stress disorder (PTSD) compared to those without depression. Similar studies conducted in Greece (87), Indonesia (15), and Ethiopia (28) supported this finding. The reason for this might be due to the fact that individuals with depression are more likely to have encountered traumatic events, which subsequently increases their risk of developing PTSD (88). Another explanation for this finding might be due to individuals who have currently depression and may have a previous history of this depression or other psychological problems, both of which can increased the risk of PTSD (89, 90). This finding implies implementing routine mental health screenings to identify students who may be experiencing depression. This can be done through surveys, questionnaires, or discussions with school counselors or mental health professionals. It is also advisable to work closely with mental health professionals, including therapists, psychologists, and psychiatrists, to develop comprehensive treatment plans for students with depression and PTSD. This may involve coordinating care, sharing information, and ensuring continuity of services. Additionally, any underlying traumatic experiences that may be contributing to both depression and PTSD are explored and addressed.

Students with high levels of perceived stress were three times more likely to develop post-traumatic stress disorder (PTSD) compared to those with low levels of perceived stress. This finding was supported by studies conducted in Ethiopia (31), Qatar (54), China (55), Bosnia, and Herzegovina (53). The possible explanation for this finding might be due to the fact that the negative perceptions regarding the harmful consequences of continuous threats can expedite the onset and continuation of PTSD (91). Practicing stress management programs or workshops teaches students coping skills and resilience-building techniques, including relaxation exercises, mindfulness practices, and time management strategies. Healthy lifestyle habits like engaging in regular physical activity, maintaining a balanced diet, getting adequate sleep, and practicing self-care techniques to help reduce stress levels are also promoted.

### Limitations of the study

This study is conducted with some limitations. One of the limitations of this study was that there may be recall bias since the study collected retrospective data of 12 months. Because of limited resources and funding, using self-report, sometimes adolescents may exaggerate their responses and report more severe symptoms; this may overestimate the finding of this study. Adolescents repeatedly discuss their experiences with each other, and this sharing of stories might influence their reports of their own experiences.

## Conclusions and recommendations

This study revealed that around two out of five high school students in the sample had experienced post-traumatic stress disorder. The independent variables that affect post-traumatic stress disorder among high school students are depression, high perceived level of stress, poor social support, witnessing the murder of family members or friends, and being in a war fighting situation. Therefore, we recommend that the Ministry of Education shall work with the Ministry of Health to include mental health professionals in addition to counseling psychologists and implement mental health services in high schools. They should implement mental health services, including the early detection of students at risk of PTSD, such as those who have depression, a high level of perceived stress, been exposed to war, or witnessed the murder of family members or friends. Providing social support to students is essential to prevent PTSD.

## Data availability statement

The raw data supporting the conclusions of this article will be made available by the authors, without undue reservation.

## Ethics statement

All procedures were conducted according to the ethical standards of the Declaration of Helsinki. The study was approved by the Institutional Review Board (IRB) of the University of Gondar. Information about the study was explained to each study participant in the information sheet. Written informed consent from the study participants who were greater than 18 years old and assent from those less than 18 years old from parents/caregiver/guardians were obtained. The right to refuse or discontinue participation at any time and the chance to ask anything about the study was given. The privacy and confidentiality of the study participants’ information were kept at every stage of data processing by not including any personal identifiers in the questionnaire. Students were not forced to participate and receive any monetary incentive, and it was solely voluntary-based.

## Author contributions

MK: Conceptualization, Data curation, Methodology, Supervision, Validation, Visualization, Writing – original draft, Writing – review & editing. SF: Conceptualization, Data curation, Methodology, Supervision, Validation, Visualization, Writing – review & editing. TA: Conceptualization, Data curation, Methodology, Supervision, Validation, Visualization, Writing – review & editing. NT: Conceptualization, Data curation, Methodology, Supervision, Validation, Visualization, Writing – review & editing. AZ: Conceptualization, Data curation, Supervision, Validation, Visualization, Writing – review & editing. GK: Conceptualization, Data curation, Methodology, Supervision, Validation, Visualization, Writing – review & editing. BA: Conceptualization, Data curation, Methodology, Supervision, Validation, Visualization, Writing – review & editing. ES: Conceptualization, Data curation, Methodology, Supervision, Validation, Visualization, Writing – review & editing.
